# Association Between Unmet Needs in Health Care and Social Services and Exposure to Violence Among Parents

**DOI:** 10.1007/s10995-024-04021-2

**Published:** 2024-11-29

**Authors:** Marianne Sipilä, Mika Helminen, Tuovi Hakulinen, Eija Paavilainen

**Affiliations:** 1 Department of Health Sciences, Faculty of Social Sciences, Tampere University, Tampere, Finland; 2 Department of Welfare Epidemiology and Monitoring, Unit of Register-Based Research and Modelling, National Institute for Health and Welfare (THL), Helsinki, Finland; 3 Tays Research Services, Tampere University Hospital, Tampere, Finland; 4 Wellbeing Services County of Pirkanmaa, Pirkanmaa, Finland; 5 Etelä-Pohjanmaa Welfare Services County, Seinäjoki, Finland

**Keywords:** Violence, Unmet service need, Health care service, Social service, Parents

## Abstract

**Objective:**

Existing research has shown that parental exposure to violence has negative consequences on health outcomes, but the effect of such exposure on unmet health care and social service need is unknown. This study aims to investigate the association between unmet health care and social services and parental violence exposure among parents with children.

**Study Design:**

This study used the data of 6289 parents aged 18–60 years who had at least one child under 18 years living in the same household. Parental violence exposure was measured. Unmet child and adult health care and social service need was operationalized through questions on the services needed, those that had not been received, and those that were considered inadequate.

**Results:**

Parents who experienced any kind of violence had more unmet service need. There were more women among parents with violence experience (65.4%) than those with no violence experience (51.9%). Violence experience increased the odds of unmet need for general adult healthcare services (OR 2.02, CI 1.64–2.57), maternity and child health clinics (OR 2.52, CI 2.00–3.18), family guidance clinics and home help (OR 2.38, CI 1.60–3.54), mental health or child welfare services (OR 2.05, CI 1.52–2.75), and school health care (OR 1.99, 1.50–2.65). After adjusting for sociodemographic factors, the associations between exposure to violence experience and unmet needs for healthcare and social services remained statistically significant.

**Conclusion:**

Violence in close relationships profoundly impacts health and well-being. By addressing unmet health care needs and supporting parents, we can break the cycle of violence and promote better mental health outcomes. Preventive policies and early interventions are essential to mitigate the consequences of violence in families.

## Significance

What is Already Known?

What this Article Adds?

It has been suggested that parents’ violence experience has an independent impact on service need. However, studies addressing the impact of any violence experience from an intimate partner or other persons in a close relationship on healthcare and social service use have shown varying results, but it has been demonstrated that violence experience impairs the use of health services.

This work, extensively examines several child and family services, highlights the need to invest in health-care and social services for parents with violence experience and with children. Service options should be sensitive to parents’ needs while offering child, adult, or family-based support. Even different types and levels of services do not necessarily help parents with any kind of violence experience find adequate assistance, but such efforts could reduce unmet child healthcare needs in vulnerable families.

## Introduction

Violence in close intimate and family relationships (VCR) encompasses various forms, including intimate partner violence, family violence, and domestic violence. VCR is an important and ongoing serious public health issue that significantly affects the health and well-being of individuals across social classes, cultures, ages, genders, and communities (Devries et al., 2013; Sardinha et al., 2022; World Health Organization, 2014). However, women are disproportionately targeted, and certain population groups are especially vulnerable to VCR. Understanding the impact of VCR is crucial for promoting gender equality, safety, and overall well-being.

### Health Impact of Violence in Close Relationships

Research indicates that the health impacts of violence are worse for women and children than for adult men (e.g., Hughes et al., 2017; Butchart et al., 2015). Violence e can continue among both adults and children as a result of negligence, maltreatment, or assault. VCR may cause acute or sustained stressors or other risk factors that may lead to detrimental physical or mental health end-results (Rantanen et al., 2022). Previous studies have suggested that children exposed to violence may suffer direct or indirect harm in families where VCRs is common. (e.g., Moraes et al., 2016; Yount et al., 2011). Adverse childhood experiences can affect their growth and development (Judd et at., 2023; Hughes et al., 2017; Klobben et al., 2015), and witnessing violence among adults in close relationships has negative consequences during early childhood (Letourneau et al., 2011) and up to adolescence (Lepistö et al., 2011); this can lead to children not receiving the treatment they need (King & Khanijahani, 2020).

### Unmet Health Care Needs

Understanding unmet health care needs is critical. International research stresses the importance of assessing these needs (e.g., Allin et al., 2010; Allin & Masseria, 2009; Bryant et al., 2009; Shemeikka et al., 2014). Unmet needs arise when individual*s* do not receive available and effective treatment. The accessibility of health services is complicated and depends on many factors reaffiliates to the national health system. Factors affecting access include age, education, socioeconomic status, past health care experience, perceived benefits, quality of care, and health literacy, (Allin et al., 2010; Allin & Masseria, 2009; Bryant et al., 2009; Lövestad et al., 2021; Shemeikka et al., 2014; Yang et al., 2019.

### Gender, Sociodemographic Factors and Service Utilization

Gender significantly predicts unmet health needs. Studies have shown that women are the main victims of violence (e.g., Manderbacka et al., 2018) and that experiencing VCR adversely affects women’s quality of life and increases their psychological distress and that experiencing VCR adversely affects women’s quality of life and increases their psychological distress (Hisasue et al., 2020). The pattern of victimization is associated with both whether survivors seek help and what kind of help they obtain (Kaukinen et al., 2013; Cho et al., 2020). Women seek formal health services less frequently (Bryant et al., 2009) than men (Cho et al., 2020).

Research highlights significant variations in service use based on sociodemographic factors. Need and social support factors have been associated with unmet need among young adults (Marshall, 2011). Young adults are the least likely to regularly consult a primary care doctor (e.g., Lövestad et al., 2021). Education can be a consistent predictor of help-seeking (Kaukinen et al., 2013). Women, especially those with greater educational achievement, are more likely to seek treatment from a variety of sources, including family, friends, and professionals (e.g., Hyunkag et al., 2020). Visits to primary healthcare general practitioners (GP) or outpatient care and unmet needs were more common among adults with less than high school-level education and those with subsistence or living difficulties. Unemployed adults also have unmet needs for physician services. (Manderbacka et al., 2013; OECD, 2013.) Some studies have argued that there is an association between economic hardship and violence exposure (e.g., Golden et al., 2013; Lucero et al., 2016). Individuals with lower education levels and those facing economic hardship are more prone to experiencing unmet needs (Palm et al., 2016; Hyunkag et al., 2020).

### Mental Health and Education

Previous studies have suggested that mental health care service for abused women are underutilized (Lipsky & Caetano, 2007; Palm et al., 2016; Fagerlund et al., 2022). Education level affects both mental health issues and unmet needs for general practitioner services (Fagerlund et al., 2022).

Individuals with less of education experience mental health issues and unmet needs for GP services more often (Yang et al., 2019). In Kaukinen et al. (2013), young women frequently reported poor mental health (Palm et al., 2016), whereas older women sought help from mental health and social service providers and called the police when needed (Kaukinen et al., 2013). Compared with those who faced only one type of violence pr none at all, women who experienced multiple forms of victimization were more likely to engage in risky alcohol use, have less educational achievement, and lack social support (Palm et al., 2016). 

### Enhancing Coping Skills and Wellbeing

Mothers who had experienced violence were more likely not have seen a doctor when needed and have not had a child health visit in the previous twelve months (King & Khanijahani, 2020). Mothers impacted by violence expressed a desire for emotional and affirmational support; they needed someone to listen to them, validate their feelings, and reassure them that their choice to leave the perpetrator was justified. Peer support or mentorship programs were also needed (Letourneau et al., 2011). Violence in a partnership can lead to estrangement, reduced self-esteem, and a diminished sense of self-worth. Increased support and coping skills can positively influence psychological well-being. Services can empower individuals to develop alternatives to abusive relationships (Coker et al., 2003).

### Legislative and Service Frameworks

In Finland, legislative frameworks such as the Health Care Act (1326/2012, §§ 15–17) and Government Decree 338/2011 mandate regular health checkups for children and adolescents. Finland’s health care system, which includes municipal primary health care services, occupational health care, and national health insurance, takes a comprehensive approach to addressing health needs, with high accessibility and low cost (Health Care Act, 2012; Keskimäki et al., 2019).

Violence in close intimate and family relationships appears to be associated with a person’s social, health, and emotional support. We hypothesize that parents with children who are exposed to violence are more likely to have unmet health care and social service needs.

## Method

### Participants

This study utilized data from the population-based cross-sectional Finnish Regional Health and Well-Being Study (ATH) conducted from 2012 to 2013. A random sample stratified by age and region was drawn from the Finnish Population Registry, encompassing 65,000 individuals from continental Finland. The questionnaires were mailed along with an informational letter. From the 32,633 responses received (53%), 6,289 participants were selected based on the following criteria: aged 18–60 years, with at least one child under 18 years living in the same household. We used information from the self-administered questionnaire in which parents were asked about their sociodemographic background and their use and experience of health care and social services.

### Measures

#### Explanatory Variable

This study used the WHO’s definition of VCR as physical, sexual, and emotional violence by an intimate partner or other persons in a close relationship (e.g., ex-partner, other family member, friend, acquaintance, or colleague) during the 12 months prior to completing the survey (Krug et al., 2002). The response categories regarding the perpetrators of such violence were “no one,” “by a current partner,” and “by another well-known person.” One of the response categories, “an unknown person” (*n* = 247), and respondents (*n* = 22) who select more than one option for the violence perpetrators were excluded. We derived a dichotomous indicator of VCR status coded “0” for parents who had no violence experience and “1” for those reporting VCR at any time. Various types of violence experienced by parents were reported more closely in Sipilä et al. (2018).

#### Outcome Variables

The need for services was measured with 15 questions on different types of health care and social services for families with children, of which the variable “need for service” was separately coded (the respondents had used at least one of 16 services). The respondents were asked “Have you been adequately provided with the following health care services over the past 12 months?” The services were as follows: visits to a general practitioner (GP) at a health center, visits to a nurse at a health center, occupational health care, social worker services, social assistance, financial and debt counseling, mental health services, substance abuse services, maternity and child health clinics, children’s municipal daycare, afternoon care for schoolchildren, home services for families with children or other family members with special needs, child guidance clinics and family counseling clinic, school health care, mental health services for children and adolescents, and child welfare services. The respondents could choose one of four options to answer this question: (1) “I have not needed it”; (2) “I would have needed it but did not receive the service”; (3) “I have used the service, but it was not adequate”; and (4) “I have used the service, and it was adequate.” Those parents who reported having had no need for health and social services were excluded from the analyses. The variable “need for services” included the response options 2, 3, and 4, with options 2 and 3 used to measure the unmet needs for health and social care services. Unmet needs for health and social care services in the past 12 months was the outcome variable examined in this study and was measured as a binary variable (0 = “no unmet needs” vs. 1 = “unmet needs”). This is a summary classification of parents’ self-perceived unmet needs for services in the past 12 months.

The service variables were recategorized into eight groups. In the case of small cells, the categories were combined. The services for parents were general adult health care services (visits by appointment to GPs and nurses or occupational health services; the latter would be to prevent work-related illnesses and accidents and promote employees’ work capacity and functioning; KELA, 2020), mental health or substance abuse services, and social and economic support (visits by appointment to social workers, social assistance, or financial and debt counseling). The services for children and families included maternity and child health clinics, daycare (children’s municipal daycare or afternoon care for schoolchildren), family guidance clinics and home help services (home services for families with children or family work, or child guidance clinics and family counseling clinics), school health care services (school health care, school counsellor, or school psychologists), and mental health or child welfare services for children (mental health services for children and adolescents or child welfare services).

### Sociodemographic Factors

The independent variables included parents’ sociodemographic characteristics which may have influenced access to or knowledge of primary health care or social care services. The sociodemographic variables assessed in this analysis included gender (male or female), parent age (18–60 years), education level (high school or less, i.e., a maximum 9 years of education, vs. post high school education, i.e., 10 or more years), employment status (yes/no) and marital status (married or cohabiting). The numbers of children aged less than 7 years and those aged 7–17 years living in the parents’ households are presented in Table 1.

**Table 1:** Sociodemographic characteristics of parents with violence or not violence experience of adults aged 18–60 years and with a least one child under 18 years. Weighted values are presented

Characteristic	Violence experience in close relationship		No violence experience		p*
	*n*	%	*n*	%	
Gender					<0.000
Women	337	65.4	3755	51.9	
Men	178	34.6	3482	48.1	
Age (mean)	37.9		39.4		0.171
Relationship Status					<0.000
Unmarried or no relationship	116	22.7	756	10.5	
Married or cohabiting	396	77.3	6472	89.5	
Level of Education					0.056
High school or less	243	54.0	3147	48.8	
Post high school	207	46.0	3301	51.2	
Employment Status					0.057
Not employed	143	28.3	1707	23.8	
Employed	362	71.7	5463	76.2	
Children in household at least one child	265	51.4	3687	50.9	0.850
Aged <7 years					
Children in household at least one child	353	68.5	4825	66.5	0.462
Aged 7–17 years					

*p-value from chi-squared test or t-test

### Statistical Analyses

Statistical analysis of the survey results was performed via SPSS 22.0 for Windows (SPSS Inc., Chicago, IL, USA). Weights were applied in the analyses to account for the sampling design and nonparticipation ensuring that the results accurately reflected the Finnish adult population. Inverse probability weights (IPWs) were used in the analyses to adjust for differences in selection probability, reduce bias due to nonparticipation, and provide nationally representative results (Seaman & White, 2013; Härkänen et al., 2014). The values for the weight variable were determined via register-based data for the entire sample, considering age, gender, marital status, education, and employment. Previous studies have indicated that the IPW method can increase the accuracy of population survey result (Carkin & Tracy, 2015; Härkänen et al., 2014).

The distribution of the data is shown as percentages and means. The analyses assessed the bivariate relationships between our outcome variables (services) and VCR status and the other covariates of interest (gender; age of parents; education level, i.e., high school or less vs. post high school education), marital status (married or cohabiting), and employment status. Bivariate logistic regression analyses were used to test the associations between unmet needs and all the covariates, including VCR status. Differences in model estimates between gender were tested using interaction terms. In addition, multivariable models including all covariates (gender, age, marital status, education, employment, and VCR status) were assessed. Adjusted odds ratios are reported together with robust standard errors and 95% confidence intervals (CIs) for weighted values. Variables were considered significant if the p-value was less than 0.05.

## Results

### Descriptive Statistics

A summary of the parents’ sociodemographic characteristics is presented in Table 1. There were more women among parents with violence experience (65.4%) than among those who had not experienced any VCR (51.9%). The mean age was 37.9 years among parents with violence experience and 39.4 years among those with no such experience. Parents with violence experience were more likely to be unmarried or not in a relationship (22.7%) than parents with no VCR (10.5%). As many as 51.4% of parents with violence experience were living with children under 7 years of age, and 68.5% were living with children aged 7–17 years.

### Unmet Need for Health Care and Social Services

Among all the respondents, 8.2% of the women (*n* = 337/4092) and 4.9% of men (*n* = 178/3660) reported that they had experienced some kind of violence in the previous 12 months. Table 2 presents the percentages of unmet service needs for parents with or without violence experience. The most unmet need for adult services during the year by parents with VCR was for general adult health care services (47.4%). There were unmet needs for social and economic support by 17.7% of parents with VCR, and 8.9% had unmet needs for mental health and substance abuse services. A majority of the parents with VCR reported no need for social and economic support or mental health and substance abuse services (70.5% and 82.9%, respectively). The most unmet need for services for children and families among parents with VCR were for maternity and child health clinic services (41.8%). One-fifth of the parents with VCR had unmet needs for school health care services, whereas 15.2% had unmet needs for family guidance clinics and home help services. A minority of unmet service needs of the parents with VCR were for daycare and mental health or child welfare services (both 11.3%).

**Table 2:** Services needed among parents with or without violence experience, weighted analyses

Charatcteristics	Service need among parents with violence experience						Service need among among parents with no violence experience					
	Unmet need		Have used, service was adequate		No need		Unmet need		Have used, service was adequate		No need	
	*n*	%	*n*	%	*n*	%	*n*	%	*n*	%	*n*	%
Services for parents												
General adult health care^1^	242	47.4	219	42.9	49	9.7	2012	28.4	3739	52.8	1331	18.8
Mental or substance abuse^1^	45	8.9	42	8.2	423	82.9	131	1.8	249	3.5	6774	94.7
Social and economic support^1^	90	17.7	60	11.8	360	70.5	420	5.9	362	5.1	6328	89.0
Services for children												
Maternity and child health clinic^2^	209	41.8	236	47.3	58	11.3	1458	21.2	4156	60.3	1274	18.5
Daycare^3^	58	11.3	186	36.2	270	52.5	419	5.9	2230	31.2	4499	62.9
Family guidance clinic services^3^	77	15.2	80	15.6	353	69.2	364	5.1	896	12.6	5859	82.3
School health care^4^	102	20.0	177	34.5	232	45.4	738	10.4	2607	36.6	3779	53
Mental health or child welfare services^3^	58	11.3	55	10.7	400	78	189	2.6	238	3.3	6722	94

^1^ Among those who have children at least <7 or 7–17 years

^2^ For children <7 years

^3^ Children <7 or 7 -17 years

^4^ Children 7–17 years

### Regression Results for Unmet Needs

Regression models were created to help improve our understanding of the parents’ unmet needs. Table 3 presents the binary logistic regression results for the associations among parents’ violence exposure, demographic variables, and unmet need for health care and social services. Violence experience was a significant factor in the unmet needs for general adult health care services (OR 2.02, CI 1.64–2.57), maternity and child health clinics (OR 2.52, CI 2.00–3.18), family guidance clinics and home help (OR 2.38, CI 1.60–3.54), mental health or child welfare services (OR 2.05, CI 1.52–2.75), and school health care services (OR 1.99, 1.50–2.65). These binary analyses were also performed separately for women and men, but there were no statistically significant differences (p > 0.05) in other sociodemographic variables or in VCR between genders.

**Table 3:** Odds ratio (OR), and 95% confidence intervals (CI) for unmet service needs among those who have used this services, weighted bivariate analysis (*N* = 6289)

Variables	General adult health care^a^ (*N* = 6140)	Mental or substance abuse^a^ (*N* = 488)	Social and economic support^a^ (*N* = 967)	Maternity and child health clinic^b^ (*N* = 6237)	Daycare^b^ (*N* = 2982)	Family guidance clinic and home help^b^ (*N* = 1470)	School health care^b^ (*N* = 3738)	Mental health or child welfare services^b^ (*N* = 566)
	OR (95% CI)	OR (95% CI)	OR (95% CI)	OR (95% CI)	OR (95% CI)	OR (95% CI)	OR (95% CI)	OR (95% CI)
Gender								
Female	1.37 (1.21–1.54)***	1.00 (0.62–1.61)	0.77 (0.52–1.06)	1.13 (0.98–1.29)	0.82 (0.65–1.03)	1.72 (1.31–2.25)***	1.15 (0.96–1.38)	0.80 (0.53–1.22)
Male	1.0	1.0	1.0	1.0	1.0	1.0	1.0	1.0
Age of parents	0.98 (0.98–0.99)***	0.99 (0.97–1.02)	0.99 (0.98–1.01)	1.02 (1.01–1.02)***	0.98 (0.96-1.00)	1.01 (0.99–1.02)	1.01 (0.99–1.02)	1.01 (0.99–1.02)
Marital status								
No relationship	1.35 (1.11–1.63)**	0.91 (0.53–1.57)	0.63 (0.45–0.89)**	1.19 (0.95–1.48)	0.95 (0.62–1.47)	1.71 (1.14–2.58)**	0.92(0.70–1.22)	0.93 (0.69–1.24)
Married or cohabiting	1.0	1.0	1.0	1.0	1.0	1.0	1.0	1.0
Education level								
High school or less	1.39 (1.23–1.57)***	1.05 (0.67–1.65)	1.25 (0.87–1.79)	1.08 (0.94–1.23)	1.47 (1.16–1.87)*	0.91 (0.69–1.19)	1.00 (0.84–1.19)	1.01 (0.85–1.21)
Post high school	1.0	1.0	1.0	1.0	1.0	1.0	1.0	1.0
Employment status								
No	1.76 (1.53–2.04)***	1.21 (0.76–1.94)	1.00 (0.73–1.39)	1.14 (0.98–1.32)	1.24 (0.94–1.64)	1.32 (1.00-1.75)	1.25 (1.01–1.56)*	1.23 (0.98–1.54)
Yes	1.0	1.0	1.0	1.0	1.0	1.0	1.0	1.0
Violence experience								
No	2.02 (1.64–2.57)***	2.06 (1.17–3.63)*	1.29 (0.84-2.00)	2.52 (2.00-3.18)***	1.66 (1.12–2.46)*	2.38 (1.60–3.54)***	1.99 (1.50–2.65)***	2.05 (1.52–2.75)***
Yes	1.0	1.0	1.0	1.0	1.0	1.0	1.0	1.0

^*^p < 0.05, **p < 0.01, ***p < 0.001

^a^Adult services

^b^Children and family services

Table 4 presents the multivariable models of the likelihood of unmet need for adult or in children and family services among parents who had or had not experienced any kind of violence. Violence experience remained a significant factor unmet needs for general adult health care services, maternity and child health clinics, family guidance clinics and home help, and school health care services. Unmet need for mental health and child welfare services did not remain significant. We detected increased odds of self-reported unmet needs for adult mental health and substance abuse services (OR 2.16, CI 1.20–3.91). Gender, education level, and employment status were associated with unmet needs for general adult health care services. Age was not a significant factor in unmet needs or any services.

**Table 4:** Multivariable models of likelihood of unmet need for adult^a^ or children and family^b^ services of parents who had or had not experienced any kind of violence, weighted analysis (*N* = 6289)

Variables	General adult health care^a^ (*n* = 4408)	Mental or substance abuse^a^ (*n* = 314)	Social and economic support^a^ (*n* = 515)	Maternity and child health clinic^b^ (*n* = 4344)	Daycare^b^ (*n* = 2007)	Family guidance clinic and home help services^b^ (*n* = 918)	School health care^b^ (*n* = 2734)	Mental health or child welfare services^b^ (*n* = 339)
	OR (95% CI)	OR (95% CI)	OR (95% CI)	OR (95% CI)	OR (95% CI)	OR (95% CI)	OR (95% CI)	OR (95% CI)
Female	1.31 (1.14–1.50)***	0.89 (0.50–1.57)	0.83 (0.57–1.22)	1.09 (0.94–1.27)	0.82 (0.63–1.06)	1.50 (1.10–2.05)*	1.13 (0.93–1.38)	0.83 (0.51–1.35)
Age of parents	0.99 (0.98-1.00)	0.99 (0.97–1.02)	1.00 (0.98-1-02)	1.08 (0.85–1.37)	0.99 (0.97–1.01)	1.02 (1.00-1.04)	1.01 (1.00-1.02)	0.98 (0.95–1.02)
No relationship	1.09 (0.88–1.35)	0.75 (0.40–1.41)	0.70 (0.47–1.04)	1.06 (0.92–1.22)	0.94 (0.58–1.53)	1.68 (1.03–2.74)*	0.88 (0.65–1.20)	0.92 (0.52–1.66)
High-school or less	1.36 (1.20–1.55)***	1.08 (0.66–1.76)	1.20 (0.82–1.75)	1.06 (0.92–1.22)	1.35 (1.04-1-75)*	0.84 (0.63–1.12)	1.00 (0.82–1.19)	0.91 (0.58–1.45)
Not employed	1.39 (1.18–1.63)***	1.33 (0.80–2.19)	0.98 (0.68–1.41)	1.13 (0.95–1.35)	1.22 (0.86–1.67)	1.21 (0.87–1.67)	1.21 (0.95–1.55)	1.13 (0.67–1.88)
Violence experience	1.69 (1.33–2.14)***	2.16 (1.20–3.91)**	1.24 (0.77–1.99)	2.45 (1.76–2.86)***	1.23 (0.79–1.92)	2.06 (1.36–3.14)***	1.98 (1.46–2.70)***	1.27 (0.72–2.24)

*p < 0.05, **p < 0.01, ***p < 0.001

^a^ Adult Services

^b^ Children and Family Services

## Discussion

This study emphasizes the associations between parental exposure to violence and unmet health care and social service needs and confirms the hypothesis that parents with children and violence experience are more likely to have unmet health care and social service needs. Consistent with earlier research, the findings from this study demonstrate that of all the violence-exposed parent respondents, nearly two thirds were female, and they were less likely to be married or cohabiting than parents with no VCR (Cho et al., 2020; Kaukinen et al., 2013; World Health Organization, 2014).

The*re *were similar numbers of children in the same households as parents with or without violence experience was similar. Our study revealed a similar trend to that recorded in Finland in 2020. According to Statistics Finland’s data, 10,800 victims of intimate partner violence and domestic violence violations were reported to authorities in 2021. Among these victims, 78.4% were adults and 75.2% were women. The victim and perpetrator lived together in the year before the statistical year, and 6% of them had a child together. A total of 50.3% of those who experienced intimate partner or domestic violence were married or cohabiting (Statistics Finland, 2021).

Compared with men, women are more likely to experience severe forms of violence and suffer more serious health consequences. (e.g., Cho et al., 2020; Kaukinen et al., 2013) However, in our study, violence was experienced by women and men and there were only minor differences between genders in terms of both unmet needs and interaction analyses with violence experience and sociodemographic factors. On this basic, we present the perspectives of parents on the experience of violence combined with the results related to service needs.

This study examined services for adults and for children and families in a health care and social service setting*s* while considering unmet service needs. The most unmet needs among parents with violence experience were for general adult health care services, including visits to GPs and nurses or to occupational health services (OR 2.02, CI 1.64–2.57). Females, individuals with a high school or lower level of education, and those who were not employed were associated with unmet general adult health care services. These results are in accordance with earlier studies of visits to GPs and unmet needs (Kaukinen et al., 2013; Lövestad et al., 2021; Manderbacka et al., 2013; OECD, 2013). In our study, parents’ age was not a significant factor, whereas Lövestad et al.’s (2021) study reported that young adults were more likely to visit a GP. In Marshall’s (2011) study, need and social support factors were associated with unmet needs among young adults, whereas in our study, only one-fifth of parents with VCR had unmet needs for social and economic support, and age was not a significant factor. This may be because economic hardship can be associated with violence exposure (Golden et al., 2013; Lucero et al., 2016).

Unexpectedly, most of the parents with VCR had no need for mental health or substance abuse services (82.9%). The association in binary analyses was weak or modest, but in the multivariate model of likelihood, it increased slightly (OR 2.16, CI 1.20–3.91). Yang et al. (2019) reported how less education and marital status are linked to unmet needs for mental health care among adult females, and that they had unmet needs for GP services more often than those in a relationship and with more education. Unemployment was not significant in our results, but Judd et al. (2023) reported that parental unemployment has been linked to an approximately 90% greater risk of child maltreatment and mental health issues in parents, this may suggest that in regard to mental health care needs, parents may refrain from seeking care despite perceiving a need for help. We were not able to examine the underlying reasons for why parents with VCR who needed help did not seek care.

Violence experience remains a significant factor in unmet needs for general adult health care services, maternity and child health clinics, family guidance clinics and home help, and school health care services. Mental health and child welfare services did not remain significant. We detected increased odds of self-reportedadult mental health and substance abuse services (OR 2.16, CI 1.20–3.91). Gender, education level, and employment status were associated with unmet needs for general adult health care services. Age was not a significant factor in any unmet needs or services.

In our study, we focused on parents’ unmet needs for services for children and families. In the binary logistic regression analysis of the associations between parents’ violence exposure and unmet needs for services, VCR was significant factor for maternity and child health clinic services (OR 2.52, CI 2.00–3.18), family guidance clinics and home help, and school health care services. In contrast, in King and Khanijahanis’ (2020), mothers with VCR and with children were more likely not to have seen a doctor when needed and not have had a well-child visit in the past year. In the multivariable model, violence experience remained significant for the same services. Only mental health or child welfare services did not remain significant. This was a surprising result, as it was expected that there would be a need for such services. Age was not a significant factor in any of these unmet needs for services.

At the risk of stating the obvious, one might say that parents’ experiences of violence leads to need for child and family services. As a result, the children of parents who have experiences violence may not receive the help and services they require (King & Khanijahani, 2020). Research has also revealed the harmful effects of adverse childhood experiences on health (Hughes et al., 2017) and how children’s health may be directly or indirectly decline (Moraes et al., 2016; Yount et al., 2011). Moreover the more parents who have experienced violence with VCR have a need for family support services, the greater the rates are of unmet needs for all support services (Hyuankag et al., 2020). In terms of concerns over children’s or adolescents’ emotional lives, behavior, and psychosocial development and health, these unmet needs can have negative effects on children’s growth and development in early childhood (Letourneau et al., 2011) and adolescence (Lepistö et al., 2011). Unexpectedly, in our study, unmet needs for mental health services for children and adolescents and child welfare services were not statistically significant, whereas VCR in general may cause serious or continuous risk factors that lead to physical or mental health consequences (Rantanen et al., 2022).

Although comparisons with previous research should be approached with caution, this study is the first to assess parents’ experiences of violence in intimate or family relationships across a wide range of health care and social services, making some discussion valuable. The current findings align with earlier studies indicating that parents who have experienced violence are more likely to have unmet health care and social service needs. An earlier study in Finland by Manderbacka et al. (2018) revealed that unmet needs for services were more common in women than in men, whereas in our findings, parents’ gender and age were not as significant as in other studies. In some studies, seeking help was more common among females than among males, and older survivors sought less help than younger survivors did (e.g., Cho et al., 2020). Some studies have also suggested that having a higher level of education and employment increases females’ likelihood of seeking help (Kaukinen et al., 2013). Therefore, the use of services is influenced by many supply-related factors, such as age and gender.

Research specifically examining the relationship between VCR and service utilization is limited and has focused primarily on affluent or high-income countries. (Sardinha et al., 2022; de Moraes et al., 2016). Finland has faced challenges in terms of the availability of services (Manderbacka et al., 2018), despite the Finnish Health Care Act (1326/2010) which guarantees all inhabitants equal, qualified, and adequate services regardless of their socioeconomic situation, economic condition, or other restrictive factors related to their service needs or factors narrowing health differences among demographic groups.

Access to health care and social services is often estimated by unmet service need (Manderbacka et al., 2018), but individual- and service structure- level obstacles may affect service use or needs. Campisi et al. (2024) reported that higher levels of parental stress, poorer coping mechanisms, and greater parental aggravation were associated with children with unmet health care needs. Parents who experience violence may be unwilling to seek health services to avoid the detection of violence due to fear of stigmatization, shame, or even revenge by the partner. The various types of support that parents need, such as support to meet their basic needs or informational support, include help connecting with support services. Previous research highlighted the obstacles that parents believe hinder their ability to access services for their children’s mental health issues. Parents noted insufficient information on accessing help, feeling that professionals did not listen to their worries, and encountering resistance from professionals regarding the initiation of interventions or referrals (Hansen et al., 2021). Additionally, Yule et al. (2019) highlighted issues such as inadequate services, lack of trust in providers, and poor communication as significant barriers to services. Unexpectedly, wait times for services during busy periods were not a barrier. While these findings are encouraging, we also note that even among those parents who do perceive a need for primary health care and social services, only a small portion access these services. Clearly, we need a better understanding of why parents who perceive a need for these services do not seek or are unable to obtain them. There is growing interest, at least in health services, in better understanding the interaction between service producers and users who have experienced violence (Manderbacka et al., 2013).

The focus of our study was on families with children under 18 years of age. In the Finnish services system, people move from one service delivery system to another, or they may use several services at the same time. The difference in between our findings and those of previous studies may also be explained by methodological differences. It is difficult to fully explain why these demographic factors were associated with help-seeking and unmet needs for services in the ways found in this study. In any case, violence within the family is usually a secret and a source of shame. The people around those involved are nearly always aware of it but are reluctant to intervene. Although further research is needed, our study can provide useful information for health care and social service practitioners; for example, they may want to not only develop a way to screen their patients or clients for violence experiences but also develop services for those who have health and social needs.

The results of this study must be considered in light of its strengths and weaknesses. This is a cross-sectional study, and it is not possible to establish the direction of the associations between the different health care and social services and the violence experience studied. It is positive that this survey, which was designed for research in adult populations, can be used to identify violence experience besauce no prior studies have examined the needs, both met and unmet, for services among parents with underage children. This approach suggests that violence experience may have varying impacts, might explain, for example, the controversial findings of previous studies.

## Conclusion

The results of our study suggest that parents with children and violence experience are more likely to have unmet health care and social service needs. There should be training programs for recognizing violence as a phenomenon, asking about the experience of violence, and using tools or measures for violence, as well as beginning to talk about sensitive issues, such as violence. Professionals need to know the available services in the areas where they work. Health care and social service professionals should pay attention to how parents can reach different services and how they can ensure sufficient services. Children are forced to live in difficult situations, and the worst thing for them is not experiencing the violence itself but the constant threat and fear of violence. Therefore, the intergenerational chain of violence experiences needs to be broken, and parents need to receive help and treatment from the necessary health care and social services. Such efforts would reduce unmet child health care needs among vulnerable families and enable more economical action by service providers.

## Data Availability

The data are available upon request with appropriate ethical approval.
